# Essential Roles of COUP-TFII in Leydig Cell Differentiation and Male Fertility

**DOI:** 10.1371/journal.pone.0003285

**Published:** 2008-09-26

**Authors:** Jun Qin, Ming-Jer Tsai, Sophia Y. Tsai

**Affiliations:** 1 Department of Molecular and Cellular Biology, Baylor College of Medicine, Houston, Texas, United States of America; 2 Program in Developmental Biology, Baylor College of Medicine, Houston, Texas, United States of America; University of Giessen, Germany

## Abstract

Chicken Ovalbumin Upstream Promoter-Transcription Factor II (COUP-TFII; also known as NR2F2), is an orphan nuclear receptor of the steroid/thyroid hormone receptor superfamily. *COUP-TFII*-null mice die during the early embryonic development due to angiogenesis and cardiovascular defects. To circumvent the early embryonic lethality and investigate the physiological function of COUP-TFII, we knocked out *COUP-TFII* gene in a time-specific manner by using a tamoxifen inducible Cre recombinase. The ablation of *COUP-TFII* during pre-pubertal stages of male development results in infertility, hypogonadism and spermatogenetic arrest. Homozygous adult male mutants are defective in testosterone synthesis, and administration of testosterone could largely rescue the mutant defects. Notably, the rescued results also provide the evidence that the major function of adult Leydig cell is to synthesize testosterone. Further phenotypic analysis reveals that Leydig cell differentiation is arrested at the progenitor cell stage in the testes of null mice. The failure of testosterone to resumption of Leydig cell maturation in the null mice indicates that COUP-TFII itself is essential for this process. In addition, we identify that COUP-TFII plays roles in progenitor Leydig cell formation and early testis organogenesis, as demonstrated by the ablation of *COUP-TFII* at E18.5. On the other hand, when *COUP-TFII* is deleted in the adult stage after Leydig cells are well differentiated, there are no obvious defects in reproduction and Leydig cell function. Taken together, these results indicate that COUP-TFII plays a major role in differentiation, but not the maintenance of Leydig cells.

## Introduction

Male fertility is controlled by complex interactions among the hypothalamus, pituitary gland, and testis [1]–[4]. Both androgen synthesis and reproductive capacity are tightly linked and regulated by various hormones, including luteinizing (LH) and follicle stimulating (FSH) hormones [5]. These gonadotropins respectively act on the Leydig cells which secrete testosterone, and the Sertoli cells which provide structural and nutritional signaling support for the developing germ cells [6]. The major functions of the testis include production of male gametes and secretion of testosterone [7], [8]. Testosterone is responsible for the development of the testes, maintenance of spermatogenesis, male fertility and secondary sex characteristics [9], [10]. Adult Leydig cells are the primary source of testosterone in the male, and it is well established that androgen production and fertility in the sexually mature male are dependent on the postnatal development of Leydig cells [11]. Leydig cells are derived from undifferentiated stem cells [12], [13] and then, at least some of them express Leydig cell-specific genes and thus become committed spindle-shaped progenitor Leydig cells [7]. The progenitor Leydig cells proliferate and also exhibit some aspects of differentiated function, including the expression of 3β-hydroxysteroid dehydrogenase (3β-HSD), cytochrome P450 side-chain cleavage (P450Scc) and luteinizing hormone (LH) receptors [7], [14], [15]. The development of the steroidogenic capacity of progenitor Leydig cells requires the stimulation of LH. These cells first transform into round immature Leydig cells followed by further increases in size and maturation into Leydig cells, acquiring the capacity for testosterone production [7], [14]. The mechanisms that regulate the proliferation and differentiation of Leydig cells are not completely understood, but in addition to testosterone [16], it is known that hormones such as LH [2], FSH [7], thyroid hormone [17], and estrogens [18] are involved in this process. In addition, it has been shown that in the absence of IGF-I [19] or PDGF-A [20] , there is a failure of Leydig cell maturation, and reduced capacity for testosterone production.

Chicken Ovalbumin Upstream Promoter-Transcription Factor II (COUP-TFII) is a member of the nuclear receptor superfamily [21]. It is highly expressed in mesenchyme and plays critical roles during mouse development [22]. *COUP-TFII* null mutants die before E10.5 due to angiogenesis and cardiovascular defects [23]. Conditional ablation of *COUP-TFII* in the limbs, stomach, diaphragm, uterus and endothelial cells reveal that COUP-TFII plays a pivotal role in cell growth, differentiation, organogenesis and lineage determination [24]–[30]. In addition, *COUP-TFII* heterozygous female mice develop significantly reduced fecundity, and the expression level of steroid biosynthetic enzymes such as P450Scc, 3β−HSD, and steroidogenic acute regulatory protein (StAR), were significantly reduced [31]. Indeed, those enzymes are required for testosterone biosynthesis in male mice [5].

To circumvent the early embryonic lethality and investigate the postnatal function of *COUP-TFII*, a tamoxifen inducible *CAGG-Cre-ER^TM^* mouse line [32] was used to delete *COUP-TFII* gene. The promoter driving Cre-ER^TM^ expression is a chimeric promoter of the cytomegalovirus immediate-early enhancer and chicken β-actin promoter/enhancer (CAGG). Here we report that the ablation of *COUP-TFII* at the pre-pubertal stage resulted in infertility, hypogonadism and arrest of spermatogenesis due to a defective testosterone synthesis in the null mice. We further demonstrated that COUP-TFII itself is indispensable for Leydig cell maturation, specifically from progenitor Leydig cells maturation into adult Leydig cells. Interestingly, we identified that COUP-TFII played roles in progenitor Leydig cell formation and early testis organogenesis, as demonstrated by the ablation of *COUP-TFII* at E18.5. In contrast, when *COUP-TFII* was deleted after Leydig cell differentiation was complete, no discernible phenotype was detected, suggesting that COUP-TFII is not important for the maintenance of Leydig cell function and male reproduction in the adult.

## Results

### Inducible Ablation of *COUP-TFII* at Pre-pubertal stage Leads to Infertility and Hypogonadism

**Figure 1:**
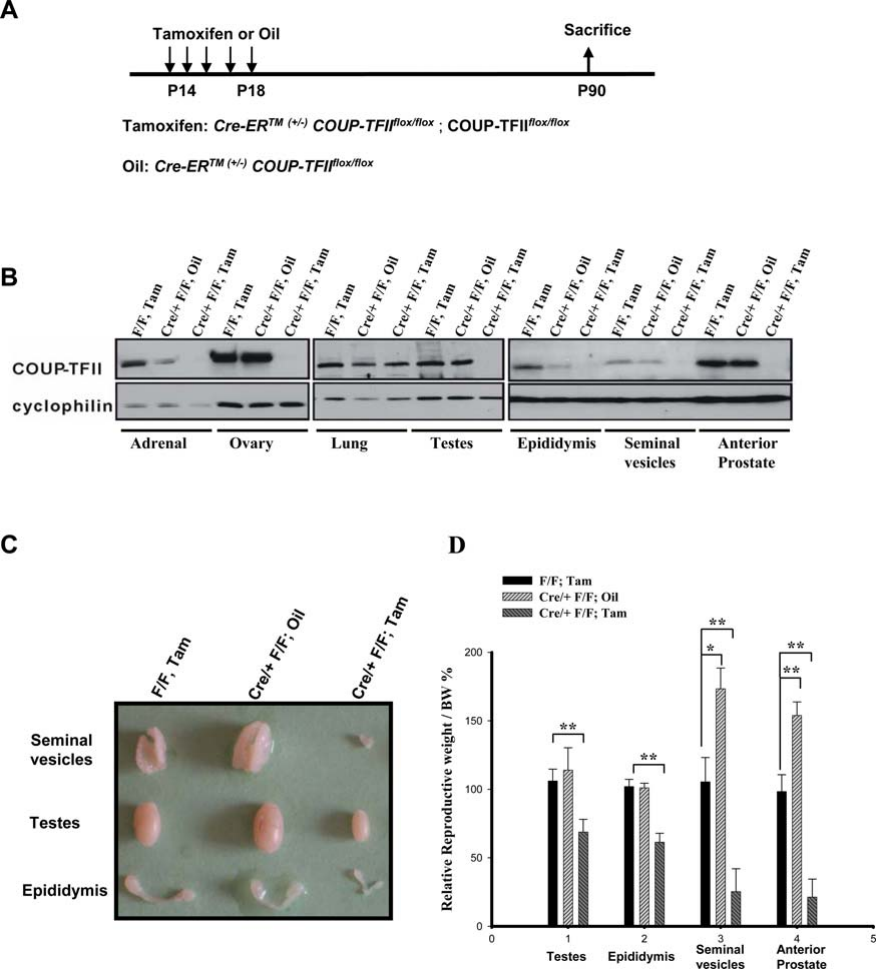
Inducible Ablation of *COUP-TFII* at Pre-puberty Stage Leads to Infertility and Hypogonadism. A) Scheme of inducible ablation of *COUP-TFII* at Pre-puberty stage. Tamoxifen or oil was intraperitonealy injected into P14 animals to induce the deletion of *COUP-TFII* gene, and mice were sacrificed at P90. B) Immunoblotting of COUP-TFII was performed to examine the deletion efficiency in mutant mice, and tissues were collected from the comparison littermates. F/F, Tam: *COUP-TF^flox/flox^* treated with tamoxifen: Cre/+ F/F, Oil: *Cre-ER^TM (+/−)^ COUP-TFII^flox/flox^* treated with oil and Cre/+ F/F, Tam: *Cre-ER^TM (+/−)^ COUP-TFII^flox/flox^* treated with tamoxifen. C) The photograph depicts the appearance of male reproduction organs from 3-month-old littermate. D) Relative weight of reproduction organs normalized with body weight. Results are expressed as the mean (±SD) of the ratios for each genotype. Statistical comparison was done with a student test. * P<0.05, ** P<0.01

*Cre-ER^TM (+/−)^ COUP-TFII^flox/flox^* mice were generated by crossing *CAGG Cre-ER^TM^* mice [32] with *COUP-TFII^flox/flox^* mice. To induce the deletion of *COUP-TFII* gene, one group of P14 *Cre-ER^TM (+/−)^ COUP-TFII^flox/flox^* animals were injected intraperitoneally with tamoxifen, while another group received only the corn oil carrier to serve as controls. A third group of *COUP-TFII^flox/flox^* animals received IP tamoxifen to ascertain the effect of tamoxifen independent of the activation of the Cre recombinase (Fig. 1A). After *COUP-TFII* was deleted at P14, our initial observation was that 3-month-old male mutant mice displayed hypoplastic external genitalia (data not shown), suggesting possible defects in reproduction. To test this hypothesis, tamoxifen-treated *Cre-ER^TM (+/−)^ COUP-TFII^flox/flox^* male mice and control male mice, including *COUP-TFII^flox/flox^* and *Cre-ER^TM (+/−)^ COUP-TFII^flox/+^* mice treated with tamoxifen, and *Cre-ER^TM (+/−)^ COUP-TFII^flox/flox^* treated with oil, were mated with wild-type females and the breeding capacity of each groups were monitored for 3 months. As shown in Table 1, no pups were born from females mated with the mutant males. In addition, we observed the majority of mutant mice failed to impregnate their mates, because vaginal plugs were not found after mating. In contrast, all types of control mice gave birth regularly, and the litter sizes did not differ significantly.

**Table 1:** Fertility Assessment and Epididymis Sperm Count.

	Vaginal plug	Litter No.	Sperm count/epididymis
COUP-TFII^flox/flox^ , Tam (n = 6)	(24/24)	5.7±1.2	2.73±0.79×10^7^
Cre-ER^TM (+/−)^ COUP-TFII^flox/flox^ , Oil (n = 7)	(28/28)	6.3±1.7	3.17±0.91×10^7^
Cre-ER^TM (+/−)^ COUP-TFII^flox/+^ , Tam (n = 4)	(16/16)	6.7±2.3	2.87±0.62×10^7^
Cre-ER^TM (+/−)^ COUP-TFII^flox/flox^ , Tam (n = 8)	(4/32)**	0	0.061±0.025×10^7^**

Summary of breeding studies. *Cre-ER^TM (+/−)^ COUP-TFII^flox/flox^* (tamoxifen) mutant mice were infertile, and the majority of them failed to impregnate the female mice, while both controls were normal in fertility. Tam, tamoxifen; ^**^ P<0.01

To verify the efficiency of Cre-mediated recombination and see whether there is any sporadic leakage independent of tamoxifen, major organs were isolated after the injection of tamoxifen or oil. As shown in Fig. 1B, except for the lung, *COUP-TFII* is efficiently ablated. However, in the absence of tamoxifen, “leakiness” of Cre-ER was detected in several organs such as the adrenal glands and epididymis. In contrast, the leakage was not detected in the testes, ovary, seminal vesicles or the anterior prostate. These observations were further confirmed by immunohistochemistry and qRT-PCR analysis of COUP-TFII protein and COUP-TFII mRNA, respectively (data not shown).

To elucidate the cause of infertility, we next examined the morphology of the male reproductive organs in the mutant mice. As compared to controls, *COUP-TFII* null mice displayed a hypogonadal phenotype (Fig. 1C). The testicular size was significantly decreased in null mice, and the weight was reduced about 35% (Fig. 1D). Consistently, the weight of epididymis from the null mice was about 62% of that from the controls (Fig. 1D). Comparison of tamoxifen-treated *COUP-TFII^flox/flox^* mice and oil-treated *Cre-ER^TM (+/−)^ COUP-TFII^flox/flox^* mice revealed that tamoxifen alone slightly reduced the weight of seminal vesicles and anterior prostate (Fig. 1D). In sharp contrast, both the size and weight of seminal vesicles and anterior prostate were more significantly reduced in the tamoxifen-treated *Cre-ER^TM (+/−)^ COUP-TFII^flox/flox^* animal (Fig. 1D). Based on these results, we conclude that the loss of COUP-TFII at pre-pubertal stage renders male mutant mice infertile and hypogonadal.

### Arrest of Spermatogenesis at the Round Spermatid Stage in the Null Mice

**Figure 2:**
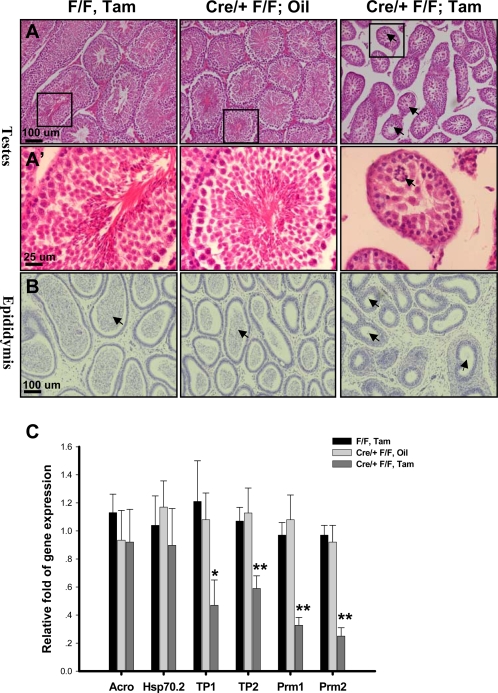
Arrest of Spermatogenesis at the Round Spermatid Stage in the Null Mice. H&E staining of paraffin-embedded testes (A, A') and epididymis (B). A' is the large magnification of the box area in the A. (C) Quantitative realtime RT-PCR analysis of the germ cell differentiation markers. RNA was isolated from 3-months-old littermates. Expression levels of each gene were normalized to the levels of the 18sRNA (n = 6). Data in (C) indicate the mean±SD. * P<0.05; ** P<0.01

In the testis, male germ cells differentiate from spermatogonia into spermatozoa by a complex process referred to as “spermatogenesis”. Mouse spermatogenetic cycle is well defined and can be subdivided into 12 stages, with each stage consisting of a specific complement of male germ cells [33]. Inspection of the seminiferous tubules in the testis from adult null mice revealed a 15 to 25% reduction in diameter (Fig. 2A and A'). Further analyses indicated that although the spermatogonia and spermatocyte appeared normal, the elongated spermatids or spermatozoa were largely absent in the tubules of null testes. In addition, we detected the presence of multinucleated cells inside the tubules of mutant testes (Fig. 2A and A'; arrow). Further examination of the caudal epididymis confirmed the spermatogenesis defects in the null mice. Mature sperm or spermatozoa was hardly observed in the null mice, with only a few dead cell bodies present in some epididymal lumen (arrow), as compared to the abundant elongated spermatids (arrow) observed in the control mice (Fig. 2B). Moreover, quantitative count assay demonstrated there were only 0.061×10^7^ sperm per epididymis in the null mice, while those in the control groups were 2.73×10^7^, 3.17×10^7^ and 2.87×10^7^ per epididymis in control mice (Table 1).

As a further step toward understanding the molecular basis of the spermatogenesis defects illustrated in Fig. 2A and B, we analyzed the expression of a number of germ cell markers by qRT-PCR. The *Proacrosin/acrosomal serine protease* (*Acro*) and *hsp70-2* gene are transcribed before the first meiotic division, and are expressed during the premeiosis phase [34]. As shown in Fig. 2C, the expression level of these genes was not significantly different among all the examined groups. Another set of genes, including *transition protein 1 (TP1)*, *transition protein 2 (TP2)*, *protamine 1 (Prm 1)*, and *protamine 2 (Prm 2)*, are expressed only during the postmeiotic phase (spermatids) [34]. We found that the expression level of all these postmeiotic phase markers were significantly reduced in the mutant testes compared to the controls (Fig. 2C). This result was consistent with our previous histological results, indicating that spermatogenesis is arrested at the round spermatid stage in *COUP-TFII* null mice.

### *COUP-TFII* Null Mice Display Leydig Cell Hypoplasia

**Figure 3:**
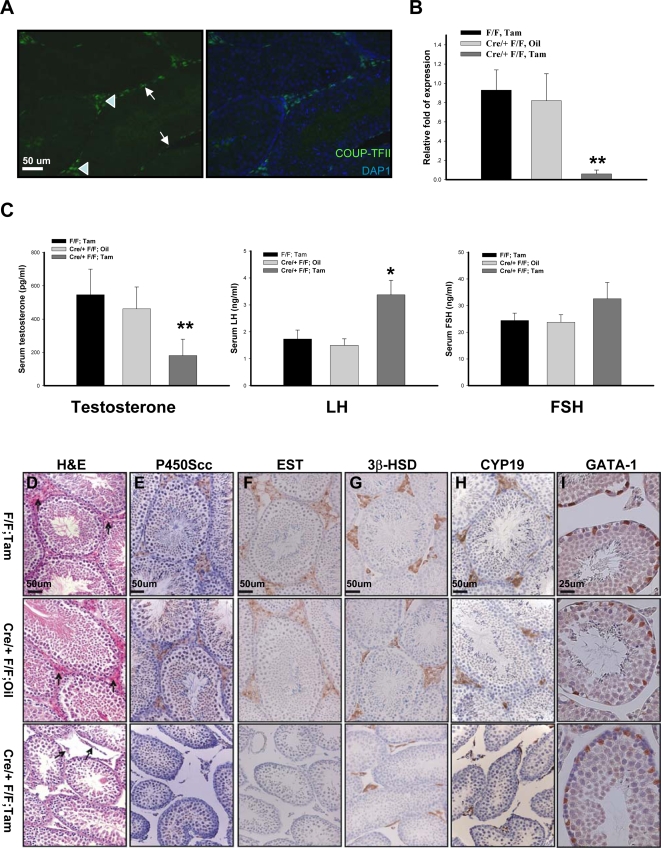
*COUP-TFII* Null Mice Display Leydig Cell Hypoplasia. (A) Immunohistological detection of COUP-TFII expression in the testes from adult wild type mice. Green, COUP-TFII; Blue: DAP1 (B) *COUP-TFII* deletion efficiency was examined by qRT-PCR. Testes were collected from the littermates. N = 6; ** P<0.01 (C) Serum testosterone, LH, and FSH levels in 3-month-old males: F/F; Tam, Cre/+ F/F; Oil and Cre/+ F/F; Tam. (n = 8, 10, and 7, respectively). (D-I) H&E staining of paraffin-embedded testes (D). Immunohistochemistry results of Leydig cell marker P450Scc (E), EST (F), 3β-HSD (G) and CYP19 (H) indicated that mutant mice display Leydig cell hypoplasia. However, Sertoli cells in the null mice are normal (I).

COUP-TFII was expressed in Leydig cells (arrowhead) and pertubular myoid cells (arrow) in adult (Fig. 3A). Furthermore, the deletion efficiency in the testes of *Cre-ER^TM (+/−)^ COUP-TFII^flox/flox^* animals was confirmed by qRT-PCR (Fig. 3B). Given the COUP-TFII expression pattern and hypogonadism phenotype, we asked whether steroid biosynthesis was defective in the mutant mice. As expected, serum testosterone concentration was 70% less in mutants compared to the controls. In addition, the serum LH level was elevated two fold in the mutant mice, presumably due to the feedback inhibitory mechanism of testosterone reduction [1], [5]. However, the increase of the FSH serum level was not significant (Fig. 3C). These results also suggested that local, but not central, regulatory mechanisms were responsible for the decreased testosterone production in the mutants.

To ascertain the cause of testosterone deficiency in the mutants, we examined the histology of Leydig cells. As shown in Fig. 3D, abundant Leydig cells were readily apparent in the testicular interstitium of adult control mice (arrow), whereas the interstitium of null testes contained very few Leydig cells with smaller sizes (arrow). Immunostaining with the Leydig cell markers such as P450Scc and EST (estrogen sulfotransferase) [15], [35] revealed barely detectable signals in the mutants (Fig. 3E and F). Whereas immunostaining of 3β-HSD and CYP19, which are also markers of Leydig cells, showed comparable intensity between the controls and the mutants in a single cell basis (Fig. 3G and H). However, these positive cells in the mutant testis appeared to be spindle-shaped and surrounded the border of the tubes, which is in contrast to the round shape and the interstitial localization in the control mice. Given the central role of Sertoli cell in the development of functional testes, we also performed GATA-1 immunostaining to examine the number and localization of Sertoli cells in seminiferous tubules from the null mice. Our results showed that Sertoli cell nuclei were located at the basal portion of the seminiferous tubules, and their number and localization were comparable among the mutants and the controls (Fig. 3I). All of these results clearly demonstrated that *COUP-TFII* null mice display Leydig cell hypoplasia, which would account for the testosterone production defect in the mutant mice.

### Hypogonadism and Spermatogenesis Defects were Rescued by Testosterone Replacement

**Figure 4:**
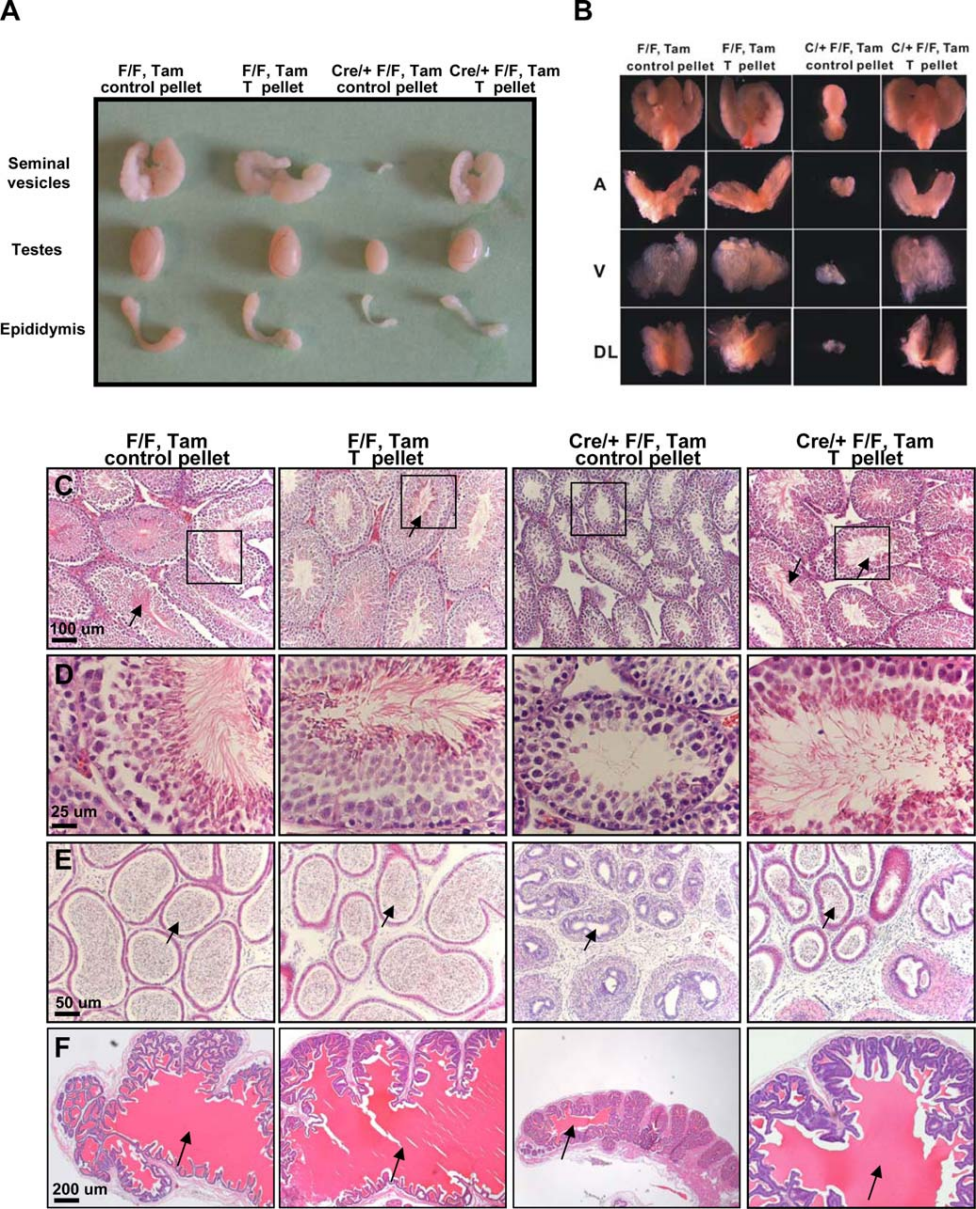
Hypogonadism and Spermatogenesis Defects were Rescued by Testosterone Replacement. Gross appearance of testes and accessory glands after testosterone treatment. The size of testes, epididymis, seminal vesicles (A) and prostate (B) grew markedly in Cre/+ F/F mice implanted with testosterone pellet compared with the null mice implanted with control pellet. H& E staining demonstrates the resumption of spermatogenesis in the null mine treated with testosterone. The elongated spermatid or spermatozoa could be observed in the mutant testes (C, D) and epididymis (E). In addition, loss of secretary protein in seminal vesicles could be observed in the rescued mice (F; arrow). A, anterior prostate; V ventral prostate; DL, dorsal-lateral prostate.

Since the pivotal role of androgens in spermatogenesis and male fertility is well established, we asked whether testosterone deficiency was the major reason for the defects in the mutant mice. *COUP-TFII^flox/flox^* and *Cre-ER^TM (+/−)^ COUP-TFII^flox/flox^* were supplemented with testosterone (as an implant), at the same time as tamoxifen injection at P14. As shown in Fig. 4A, after testosterone treatment for 45 days, the size of mutant testes was indistinguishable from those of the control mice. In addition, the hypoplastic accessory sex glands of the mutant (epididymis, seminal vesicles, and prostate) grew markedly during testosterone treatment, reaching the size of those in the control mice (Fig. 4A and 4B). Histological examination of the epididymis, seminal vesicle, and prostate confirmed that the hypogonadal phenotype was largely due to testosterone deficiency in the mutant (Fig. 4C–F). In addition, the expression of secretory protein in seminal vesicle, whose synthesis is also dependent on androgen [36], also appeared normal after testosterone treatment (Fig. 4F; arrow).

Furthermore, we observed that testosterone treatment stimulated the growth of the seminiferous tubules, which contained all stages of spermatogenic cells, including mature sperm in the enlarged lumen (Fig. 4C; arrow). In addition, resumption of full spermatogenesis in the rescued animals could be seen in the epididymis (Fig. 4E; arrow). However, the epithelial cells in the caudal region of epididymis from the rescued animals were still disorganized.

### Indispensable Roles of COUP-TFII in Progenitor Leydig cell Maturation

**Figure 5:**
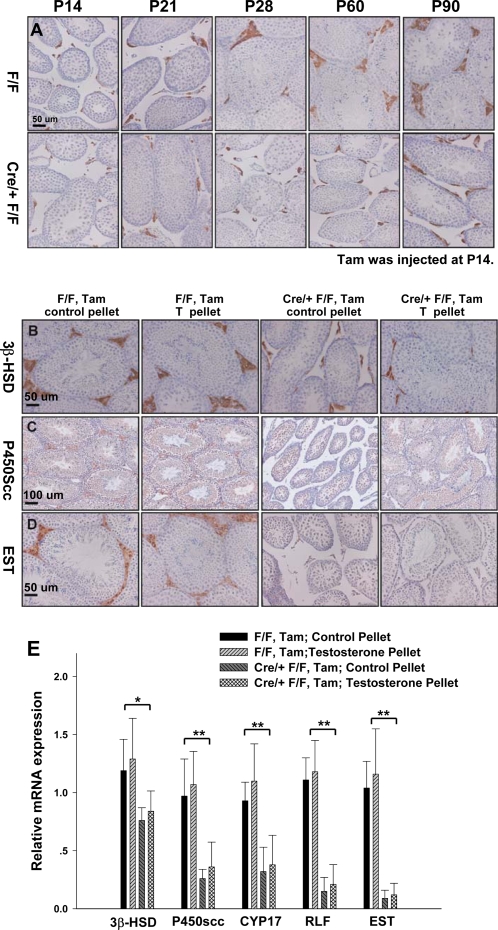
Indispensable Roles of COUP-TFII in Progenitor Leydig Cell Differentiation. A) Evaluation of Leydig cell differentiation in the mutant testes, indicated by Leydig cell marker 3β-HSD immunostaining. The samples were collected at P14, P21, P28, P60 and P90, which correspond to 0, 7, 14, 46 and 76 days after tamoxifen injection, respectively. B) Testosterone treatment could not rescue the hypoplasia of Leydig cells. There were no increases in the intensity of P450Scc (C) and EST (D) signals or localization changes of 3β-HSD (B) signals in the rescued animal. E) qRT-PCR analysis of the expression of Leydig cell marker. RNA was isolated from 3-months-old littermates implanted with control or testosterone pellet (n = 6). Expression levels of each gene were normalized to the levels of the 18sRNA. Data in (E) indicate mean±SD. * P<0.05; ** P<0.01

To address the involvement of COUP-TFII in Leydig cell differentiation, we collected testicular tissue at different time points after tamoxifen injection. At P14 just before tamoxifen injection, progenitor Leydig cells were well formed, appearing to be spindle-shaped and having a peri-seminiferous tubule localization. As expected, there was no difference in the number of progenitor Leydig Cells between *COUP-TFII^flox/flox^* and *Cre-ER^TM (+/−)^ COUP-TFII^flox/flox^* (Fig. 5A). In addition, a similar result was observed at P21, the time corresponding to 7 days after tamoxifen injection. At later stages, progenitor Leydig cells began to differentiate into mature Leydig cells in the control mice, whereas Leydig cell differentiation in the mutant was arrested at the progenitor Leydig cell stage, and did not undergo any further maturation. (Fig. 5A)

Differentiation of Leydig cell precursors into mature Leydig cells is a key step in development of the Leydig cell population [7], and androgens play a positive role in this process [16]. We asked whether defects in mutant Leydig cells were due to the absence of *COUP-TFII* itself, or the consequences of testosterone deficiency in the mutant mice. As shown in Fig. 5B–D, testosterone treatment did not improve the diminished intensity or the mislocalization of EST, P450Scc and 3β-HSD immunoreactivity. Our observations were further substantiated by the diminished mRNA level of five Leydig cell-specific genes measured by qRT-PCR. Three genes which are related to steroid biosynthesis including3β-HSD, P450Scc and CYP17, start to be expressed when the cells have committed to the Leydig cell lineage [7]. As shown in Fig. 5E, the mRNA content of 3β-HSD was only reduced by 20%, while the reduction of P450Scc and CYP17 expression levels was much more pronounced in COUP-TFII null mice. More importantly, treatment with testosterone failed to rescue the expression levels of these genes. We also examined the expression of Leydig cell differentiation markers including EST and relaxin-like factor (RLF), which are only highly expressed in fetal and mature adult Leydig cells [15]. The expression of EST and RLF was barely detectable in the mutant implanted with either control or testosterone pellets (Fig. 5E). Taken together, these findings indicated that COUP-TFII itself is indispensable for the Leydig cell differentiation process, particularly related to the step involved in maturation of progenitor Leydig cells to adult Leydig cells.

### COUP-TFII Plays Roles in Testis Organogenesis and Progenitor Leydig Cell Formation

**Figure 6:**
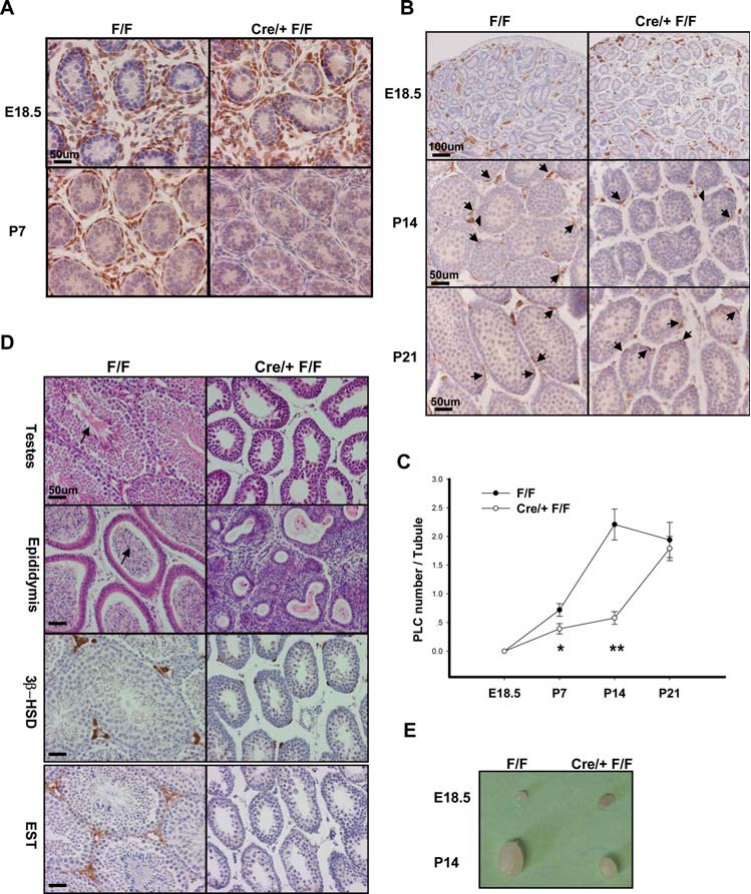
COUP-TFII Plays Roles in Testis Organogenesis and Progenitor Leydig cell Formation. A) Immunohistochemistry for COUP-TFII at embryonic 18.5 (E18.5) and P7. Tamoxifen was injected to pregnant mothers at E18.5. B) Immunohistochemistry for Leydig cell markers, 3β-HSD at E18.5, P14 and P21. Arrow indicated progenitor Leydig cells, and arrowhead was fetal Leydig cells. C) Quantitative results of progenitor Leydig cell number peri-seminiferous tubule. Data in (C) indicate mean±SD. * P<0.05; ** P<0.01. D) H& E staining of the testes and epididymis from P60 littermate of control and mutant mice. Spermatazoa was indicated by arrow. Immunohistochemistry result for Leydig cell markers, 3β-HSD and EST. E) The photograph depicts the appearance of testes from control and mutant mice at E18.5 and P14.

To examine whether COUP-TFII is important for early testis organogenesis and progenitor Leydig cell formation, the pregnant mothers (E18.5) received tamoxifen treatment to induce the deletion of *COUP-TFII* in pups. At P7, immunohistochemistry analysis was performed to examine the deletion efficiency of *COUP-TFII*. As shown in Fig. 6A, COUP-TFII was highly expressed in the mesenchymal cells of testes before tamoxifen injection (E18.5). There were comparable expression levels between the control and mutant mice. In contrast, COUP-TFII was efficiently deleted in the P7 mutant mice, whereas it was highly expressed in the control mice. In terms of progenitor Leydig cell formation, we used 3β-HSD as the Leydig cell maker combining with cell localization and spindle-like shapes to distinguish the progenitor Leydig cells (arrow) from fetal Leydig cells (arrowhead). It is known that fetal Leydig cells arises soon after testis differentiation at about E12.5 in the mouse, and are essential for masculinization of the fetus [11]. Concurrent with the postnatal appearance and differentiation of adult Leydig cells, the number of fetal Leydig cells diminishes after birth [14]. We examined the fetal Leydig cells population in E18.5 testis to ascertain the tamoxifen independent activation of the Cre recombinase, and there was no difference between the control and mutant mice. At P7 and P14, significantly less progenitor Leydig cells were observed in the mutant testis (Fig. 6B; arrow and data not shown). However, there was no clear difference at P21 (Fig. 6B; arrow). Further quantitative analysis demonstrated progenitor Leydig cell formation was delayed in the mutant testis (Fig. 6C). In addition, we also isolated the reproductive organs from P60 animals, in which COUP-TFII was knocked out at E18.5. As expected, the mutant male mice displayed spermatogenesis arrest, Leydig cell hypoplasia and hypogonadism defects. As shown in Fig. 6D, spermatozoa (arrow) was absent in the testes and epididymis of the mutant mice. Furthermore, the barely detectable signals of EST and spindle-shape positive cells of 3β-HSD indicated the failure maturation of Leydig cells in mutant testes (Fig. 6D). Interestingly, we found the size of testes harvested from the P14 mutant animal was much smaller in comparison with its littermate controls, indicating that COUP-TFII also played important roles in early testis organogenesis (Fig. 6E).

### COUP-TFII is not Essential for Maintenance of Leydig cell Function

**Figure 7:**
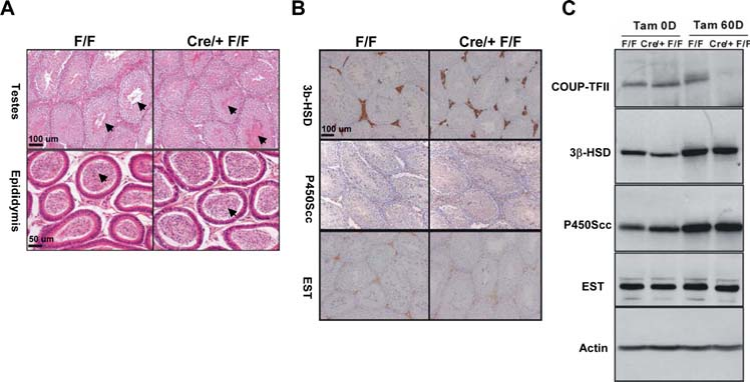
COUP-TFII is not Essential for Maintenance of Leydig Cell Function. A) Histological examination of testis and epididymis. Spermatazoa (arrow) were observed in testes and epididymis of 4-month-old control and Cre/+ F/F mice, which were treated with tamoxifen at the age of two months. B) Immunohistochemistry for Leydig cell markers, 3β-HSD, P450Scc and EST. C) Immunoblotting for COUP-TFII, 3β-HSD, P450Scc and EST. Tam OD indicates the day before tamoxifen injection when mice are two month old. Tam 60D means 60 days after tamoxifen injection.

Since COUP-TFII is also expressed in mature Leydig cells in the adult, we asked whether COUP-TFII is also essential for the maintenance of Leydig cell function. Tamoxifen was administered to induce the ablation of *COUP-TFII* gene at two months of age, when Leydig cells were fully developed. After two months of *COUP-TFII* deletion, the histology of testis and epididymis revealed that spermatogenesis was normal in the mutants (Fig. 7A). Immunohistochemistry results of 3β-HSD, P450Scc and EST showed that the expression level of these markers in the mutants were comparable with the controls. Consistent with these findings, western blot confirmed that COUP-TFII was not essential for the maintenance of Leydig cell marker genes expression in adults (Fig. 7B and 7C). As expected, the serum testosterone level in mutants was also comparable to the controls (data not shown). In addition, we also examined the fertility of mutant mice. There was no obvious difference between control and mutant mice. Based on these results, we conclude that COUP-TFII is not essential for the maintenance of Leydig cell function and male fertility in adult under normal conditions.

## Discussion

In this study, we used a tamoxifen inducible Cre-ER recombinase to delete *COUP-TFII* gene at pre-pubertal stage, and clearly demonstrated that COUP-TFII is indispensably required for progenitor Leydig cell maturation, and ultimately contributes to testosterone synthesis and fertility. In addition, we identified that COUP-TFII played roles in progenitor Leydig cell formation and early testis organogenesis, as demonstrated by the ablation of *COUP-TFII* at E18.5. In contrast, COUP-TFII is not essential for the maintenance of Leydig cell function as shown by deletion of *COUP-TFII* in adult male mice. An advantage of this mouse model was to allow us to clearly determine that COUP-TFII is important for Leydig cell differentiation, but not for the cell maintenance. Also, this temporal knockout model facilitates elaborate dissection of COUP-TFII function during each differentiation stage. Notably, we demonstrate that administration of testosterone could largely rescue the hypogonadal and spermatogenetic defects in the mutant animal. However, the Leydig cell is still dysfunctional. The results form rescued animals provide the evidence that the major function of adult Leydig cells is to produce testosterone.

In the testis, COUP-TFII is highly expressed in mesenchymal cells at early stages such as E18.5 and P14. However, during testicular organogenesis, COUP-TFII expression gradually decreases after P21, due to the reduced number of mesenchymal cells. Using this tamoxifen-inducible knockout model, we also deleted *COUP-TFII* at E18.5, and testes were collected at P7, P14 and P21. Interestingly, the size of testis in the mutants was also greatly reduced when compared to controls at all stages examined (Fig. 6D and data not shown). Since circulating testosterone decreases significantly after birth, and does not rise back before the pubertal stage [15], it is reasonable to speculate that COUP-TFII is required for testes organogenesis at the early stages (P1–P14). In terms of Leydig cells, we observed that progenitor Leydig cell formation was delayed in the mutant testis. The high expression of COUP-TFII in mesenchymal cells and the fact that progenitor Leydig cells originated from mesenchymal stem cells [12], [14] suggests that COUP-TFII may also be required for Leydig cell lineage commitment. All these observations indicated that COUP-TFII plays multiple roles in testicular development such as organogenesis, lineage determination as well as Leydig cell differentiation.

Here we present data showing the important role of COUP-TFII for the progenitor Leydig cell maturation. In the absence of COUP-TFII, progenitor Leydig cells could not achieve further maturation. To our knowledge, COUP-TFII is the first identified transcription factor intrinsic to the Leydig cell, and plays an essential role in progenitor Leydig cell maturation. However, the detailed molecular mechanisms still remain undefined. There are multiple complex signaling pathways regulating this process, involving LH [2], [3], IGF-1 [19] and PDGF signal pathways [20]. It has been shown that mouse knockout of the LH receptor leads to an arrest of Leydig cell differentiation at progenitor Leydig cell stage [3], [4]. Also, *LHβ* null mice display Leydig cell hypoplasia [2]. All these results indicate that LH signaling pathway plays roles not only in testosterone synthesis, but also in Leydig cell differentiation. The similar phenotype observed in our inducible COUP-TFII knockout mice suggested that COUP-TFII might be the downstream of LH signal pathway in the regulation of Leydig cell differentiation. Furthermore, Leydig cell hypoplasia observed in IGF-1 null mice shows that IGF-1 is important for Leydig cell differentiation. To identify downstream targets of COUP-TFII, we also examined the expression level of IGF's signaling pathway components such as IGF-1, IGF receptor as well as IGF binding protein-3 (IGFBP3) in our mutant mice. However, we did not observe any significant differences among all samples examined (data not show). As a future direction, it would be interesting and necessary to identify the downstream target genes that are regulated by COUP-TFII during Leydig cell differentiation.

Although testosterone replacement rescued the mutant hypogonadal and spermatogenetic defects, the majority of mutant mice were still infertile. Although the mating behavior was restored, only less than 20% of rescued animals were fertile (data not shown). In the rescued animals, the serum testosterone level was already increased two- to three -fold compared with the control mice. Notably, COUP-TFII is highly expressed in the stromal cells of other reproductive organs such as the epididymis, seminal vesicles and prostate at an early stage. It is possible that COUP-TFII deletion affected one or all three above organs contributing to infertility. For example, we still observed disorganized epithelial cells in the epididymides of rescued animals. Epididymal dysfunction may have affected sperm motility and male fertility. It is interesting that COUP-TFII not only impacts testosterone biosynthesis, but may also play important roles in other reproductive functions, which ultimately affects male fertility. Given that testosterone alone cannot rescue the infertility of mutant mice, we believe that COUP-TFII might be a potential drug target to treat male infertility.

In a recent study, it was reported that tamoxifen inducible *Cre-ER* line has toxicity in hematopoiesis and the increase of apoptosis in many tissues during embryonic development, and the toxicity also depends on the injection dose of tamoxifen [37]. However, we did not observe defects in fertility and spermatogenesis from *Cre-ER^TM (+/−)^ COUP-TFII^flox/+^* animal, excluding the possibility that the phenotype we observed in COUP-TFII null mice was due to Cre-ER toxicity. Not only will this study provide a new insight into the process of Leydig cell differentiation, but it also will further our understanding of the dysregulation of steroid hormone synthesis and male reproduction.

## Materials and Methods

### Animal Experiments

Generation of *COUP-TFII^flox/flo^* ^x^ mice and *CAGG-Cre-ER^TM^ mice* (Jackson Lab) has been previously described [30], [32]. Mice used in this study were of mixed background, which were backcrossed three generations C57BL/6J background (Taconic), and maintained according to the National Institutes of Health Guide for the Care and Use of Laboratory Animals. All experiments were approved by the Animal Center for Comparative Medicine at Baylor College of Medicine. Tamoxifen (Sigma) was dissolved in corn oil (Sigma) at a concentration of 10 mg/ml. To induce *COUP-TFII* deletion after birth, mice were intraperitoneally injected with tamoxifen at 1 mg per 50 g of body weight for 5 consecutive days starting at P14 or P60. For the deletion in embryonic stages, embryos received tamoxifen through blood circulation from the mothers. Pregnant mothers (E18.5) were intraperitoneally injected with tamoxifen at 2 mg per animal.

### Fertility Assay

We investigated reproductive capacities of *COUP-TF^flox/flox^* (Tamoxifen), *Cre-ER^TM (+/−)^ COUP-TFII^flox/flox^* (Oil), *Cre-ER^TM (+/−)^ COUP-TFII^flox/+^* (Tamoxifen) and *Cre-ER^TM (+/−)^ COUP-TFII^flox/flox^* (Tamoxifen) mice by mating one male with two females for 3 months. Female mice were checked for vaginal plugs each morning, and litter sizes were recorded on delivery during two successive matings.

### Evaluation of Epididymal Sperm

The caudal epididymis were removed and minced in 0.1 ml of motile buffer (120 mM NaCl, 5 mM KCl, 25 mM NaHCO_3_, 1.2 mM KH_2_PO_4_, 1.2 mM MgSO_4_ and 1.3 mM CaCl_2_). The tissues were incubated at 37°C for 5 min to allow sperm to disperse, as described previously [38]. The total sperm count was assessed in the final suspension by using a hemacytometer.

### Histology and Immunohistochemistry

For immunohistochemistry, mouse testes were fixed overnight in 4% paraformaldehyde (PFA)/PBS, dehydrated through graded ethanol, and processed for paraffin embedding. Primary antibodies used in this study are as follows: mouse monoclonal anti-COUP-TFII (Perseus Proteomics), Goat polyclonal anti–3β-HSD, Goat polyclonal anti-CYP19, rabbit polyclonal anti-GATA-1 (Santa Cruz Biotechnology), rabbit polyclonal anti-P450Scc (Chemicon), rabbit polyclonal anti-EST (Biovision). Biotinylated antibodies (Jackson ImmunoResearch,) were used as secondary antibodies, followed by horseradish peroxidase–conjugated streptavidin (Molecular Probes,), and signals were developed with 3, 3′-diaminobenzidine (DAB) substrate kit (Vector Laboratories) or tyramide signal amplification (TSA) kit (Molecular Probes). Hematoxylin was used for counterstaining in immunohistochemistry.

### Western Blot Analysis

Isolated tissues were homogenized and lysed with 1×RIPA buffer (150 mM NaCl, 10 mM Tris-Cl [pH 7.5], 0.1% SDS, 0.1% Triton X-100, 1% deoxycholate, and 5 mM EDTA). Protein concentrations were determined by the BCA protein assay system (Pierce, Rockford, IL). 20 µg of protein was loaded and separated on 4–15% polyacrylamide gels, then electroblotted onto nitrocellulose membranes. The membranes were blocked and then incubated overnight with primary antibody at 4°C, followed by incubation with HRP-conjugated second antibody (DAKO). Signals were visualized with ECL plus Western Blotting Detection System (Amersham Biosciences, UK).

### Quantitative Realtime RT-PCR

Total RNA was extracted by Trizol methods according to the manufacture protocol (Invitrogen) and reverse transcribed using TaqMan Reverse Transcription Reagents (Applied Biosystems,). Gene expression assay was performed using the ABI PRISM 7700 Sequence Detector System (Applied Biosystems). TaqMan Universal Master Mix reagents and inventoried primer/probe mixture (Applied Biosytems) were used for the reaction. Standard curves were generated by serial dilution of a preparation of total RNA, and all mRNA quantities were normalized against 18S RNA using ABI rRNA control reagents. The primers/probes used in this study as follows: *P450Scc* (Mm00490735_m1); *StAR* (Mm00441558_m1); *3β-HSD* (Mm00476184_g1); *COUP-TFII* (Mm00772789_m1), *TP1* (Mm00437165_g1); *TP2* (Mm00726979_s1); *Hsp70-2* (Mm00434069_s1); *Prm1* (Mm01342731_g1); *Prm2* (Mm03048199_m1); *RLF* (Mm01340353_m1); *EST* (Rn00820646_g1); *Acro* (Mm00496484_g1) and Eukaryotic *18S rRNA* (4319413E).

### Testosterone Replacement

Sixty-day time-release pellets containing 7.5 mg testosterone (Innovative Research America, Sarasota, FL) were subcutaneously implanted into null animals at P14 together with tamoxifen injection. The pellets represent a matrix-driven delivery system that effectively and continuously releases testosterone. Null mice for controls were implanted with control pellets. After 45 days of testosterone treatment, the animals were approximately 8 weeks older. Mice were sacrificed to recover the reproduction organs and blood.

### Steroid Hormone Assay

Total testosterone level was measured with ELISA kits (Assay Designs Inc., Ann Arbor, MI). The serum LH and FSH levels were measured with radioimmunoassay by the core laboratory of University of Virginia Center for Research in Reproduction Ligand Assay and Analysis Core.
